# Older Adults’ Perspectives on Using Digital Technology to Maintain Good Mental Health: Interactive Group Study

**DOI:** 10.2196/11694

**Published:** 2019-02-13

**Authors:** Jacob A Andrews, Laura JE Brown, Mark S Hawley, Arlene J Astell

**Affiliations:** 1 Centre for Assistive Technology and Connected Healthcare School of Health and Related Research University of Sheffield Sheffield United Kingdom; 2 Manchester Academic Health Science Centre Division of Psychology and Mental Health University of Manchester Manchester United Kingdom; 3 Ontario Shores Centre for Mental Health Sciences Whitby, ON Canada; 4 School of Psychology and Clinical Language Sciences University of Reading Reading United Kingdom; 5 Department of Occupational Sciences & Occupational Therapy Faculty of Medicine University of Toronto Toronto, ON Canada; 6 Department of Psychiatry Faculty of Medicine University of Toronto Toronto, ON Canada

**Keywords:** mental health, older adults, technology, digital technology, Internet, apps

## Abstract

**Background:**

A growing number of apps to support good mental health and well-being are available on digital platforms. However, very few studies have examined older adults’ attitudes toward the use of these apps, despite increasing uptake of digital technologies by this demographic.

**Objective:**

This study sought to explore older adults’ perspectives on technology to support good mental health.

**Methods:**

A total of 15 older adults aged 50 years or older, in two groups, participated in sessions to explore the use of digital technologies to support mental health. Interactive activities were designed to capture participants’ immediate reactions to apps and websites designed to support mental health and to explore their experiences of using technology for these purposes in their own lives. Template analysis was used to analyze transcripts of the group discussions.

**Results:**

Older adults were motivated to turn to technology to improve mood through mechanisms of distraction, normalization, and facilitated expression of mental states, while aiming to reduce burden on others. Perceived barriers to use included fear of consequences and the impact of low mood on readiness to engage with technology, as well as a lack of prior knowledge applicable to digital technologies. Participants were aware of websites available to support mental health, but awareness alone did not motivate use.

**Conclusions:**

Older adults are motivated to use digital technologies to improve their mental health, but barriers remain that developers need to address for this population to access them.

## Introduction

Late-life mental health is often overshadowed by physical health. However, mental illness is common in older adult populations, with estimates suggesting that depression is more common than dementia in later life [1]. Prevalence of depression among individuals over the age of 65 has been suggested to range from 7.2% to as high as 49%, depending on the living situation from which the sample is taken [2]. Depression is characterized by persistent feelings of low mood, lack of motivation, reduced enjoyment of daily activities, tiredness, and suicidality [3]. Over half of all cases of depression in older adults are first onset [4], highlighting the need for effective preventative strategies for this age group. Generalized anxiety disorder is also common, affecting up to 15% of older adults, with even higher rates of symptom incidence [5]. Anxiety has several symptoms in common with depression and can cause persistent and exaggerated worry, trembling, and panic attacks [3]. Both anxiety and depression are risk factors for suicide [6,7] and can negatively impact physical health and well-being. This is evident in hospital care, where older adults with mental health problems spend longer waiting for emergency room treatment, have longer hospital stays, and have higher rates of readmission than those without them [8]. In addition, the presence of mental health problems in older adults is correlated with increased health care costs, even after accounting for mental health-specific treatment costs [9].

In contrast, there is growing interest in efforts to promote positive mental health, well-being, and flourishing, including among the older adult population. Keyes defines mental health as “a syndrome of symptoms of positive feelings and positive functioning in life,” and uses the term *flourishing* to describe states of positive mental health [10]. The New Economics Foundation developed the *Five Ways to Wellbeing*, a series of tools to help individuals to improve well-being and increase flourishing. These tools comprise five types of behavior, each of which is likely to increase positive affect, according to evidence reviewed by the foundation [11]. The report specifically mentions the benefits these behaviors can have for older adults. Huppert reviewed literature on the causes and consequences of psychological well-being and concluded that actions and attitudes were likely to have a greater effect on mental state than external circumstances [12]. These accounts suggest that individuals can have a strong influence over their own mental states by engaging in behaviors known to support good mental health. However, for individuals to take responsibility for their own mental states, awareness of their present mental state and knowledge of the behaviors that may improve it are required.

Across all age groups, the potential of digital technologies for monitoring and supporting good mental health is increasingly being recognized [13]. For example, there is increasing use of digital tools for ecological momentary assessment or experience sampling of mood [14,15], as well as recording of GPS data to monitor mental health using digital technology [16]. There is also a growing range of mobile apps to suggest and facilitate activities to maintain positive mood, for example, MoodMission [17], Happier [18], and HeadSpace [19]. Older adults are also making increased use of digital technologies [20,21], particularly for recreational activities [22] and keeping in contact with people they care about [23]. However, there has been less investigation of their use of technologies to support mental health. Those studies that have explored this area have limitations. For example, while Sauve et al [24] found a significant increase in participants’ self-reported mood after playing an online educational game on the topic of well-being, they only examined one game, developed by the authors themselves, limiting generalizability to other digital tools. Similarly limited are the findings from a single case study examining online, therapist-assisted cognitive behavioral therapy, which revealed user satisfaction and positive responses to the use of technology for this purpose [25]. Given these limited results, more research is needed involving larger groups of older adults to increase the range of views expressed.

There is also a need to understand potential barriers to older adults using digital technologies for supporting mental health. For instance, many older adults perceive technology to be expensive [26,27] and often prefer the “old-fashioned” ways of doing things [28]. Prior research has also highlighted that older people can have trouble remembering how to use new technologies [27,28]. However, little is known about older adults’ attitudes toward the use of digital technologies specifically to support mental health.

To address these gaps in knowledge, this study sought to use an innovative and interactive methodology to understand what motivates and prevents the use of digital technologies to support mental health among older adults. Therefore, this study was based on the Challenging Obstacles and Barriers to Assisted Living Technologies (COBALT) [29] principle of *user as expert* [30]. According to this principle, older adult users of technology are considered the most knowledgeable on the reasons for their adoption and continued use, or abandonment, of particular technologies. This approach invites older adults to share momentary experiences of using technologies presented within the session and addresses the limitations of traditional focus groups that rely on participants recalling anecdotes from memory [31], perhaps from years ago, where subtle details of particular situations may be lost.

The COBALT approach was cocreated with older adults as a means of ensuring their equal participation in technology development and evaluation. Most older adults have no experience interacting with technology developers, but their input into developing technologies and services aimed at them is essential. The COBALT project created user-centered activities specifically to hear the voices of older adults at any and all stages of the technology development process [32]. These arose in response to older adults, technology developers, and service providers who might commission or provide technologies to older adults, identifying lack of meaningful communication as a major barrier to technology uptake. The principle of *user as expert* and some of the specific activities have been further developed in the TUNGSTEN project [33]. All parts of the interactive sessions are audio- and videotaped, meaning that views participants may only express to one or two of their peers are also captured, reducing the influence of large-group dynamics on results. In this study, each facilitated activity used a set of materials or technologies, developed to create a comfortable environment where older adults could speak freely as experts about the topic of interest, which in this case was technology and late-life mental health.

## Methods

### Design

This study used a qualitative approach to elicit older adults’ views and experiences. It sought to explore why older adults might or might not be motivated to engage with digital technologies to support their mental health. The aim was thus to develop new insights and understanding, which are key aims of a qualitative approach. The COBALT tool kit uses techniques that encourage interaction with technology within sessions, so that immediate reactions and experiences can be captured, and that respect the knowledge held by older adults regarding their motivations and experiences of using technologies.

### Participants

In total, 15 participants were recruited for the study: one group of 7 participants and one group of 8, which is in line with COBALT methodology guidelines [34]. Eligibility criteria were as follows: being 50 years of age or over, able to read (with or without glasses), able to hear (with or without a hearing aid), and having no diagnosis of cognitive impairment (self-certified). As the aim of the study was to understand views on apps and websites to maintain good mental health rather than treat mental illness, people experiencing mental illness at the time of recruitment were ineligible for the study. While several organizations describe older adulthood as beginning at age 65, we recruited adults aged 50 and older, in line with the Mental Health Foundation’s definition of later life as the period beginning at age 50 [35]. While this meant the age range was large, it ensured that views of people likely to have experienced using digital technology during their working lives were included in the study. This was to ensure the research would remain relevant to those reaching retirement age over the next 15-20 years. No upper age limit was set. Participants were recruited from two volunteer groups aimed at people aged 50 years and older and participants were advised that they did not need to have extensive knowledge of technology to take part.

### Ethics and Payment

The study was approved by the Research Ethics Committee of the School of Health and Related Research at the University of Sheffield (approval number 003140). All participants provided written informed consent before taking part. No payment was provided to the participants, although return taxi fares to the session venue were offered and refreshments were provided in all sessions.

### Materials

**Figure 1:**
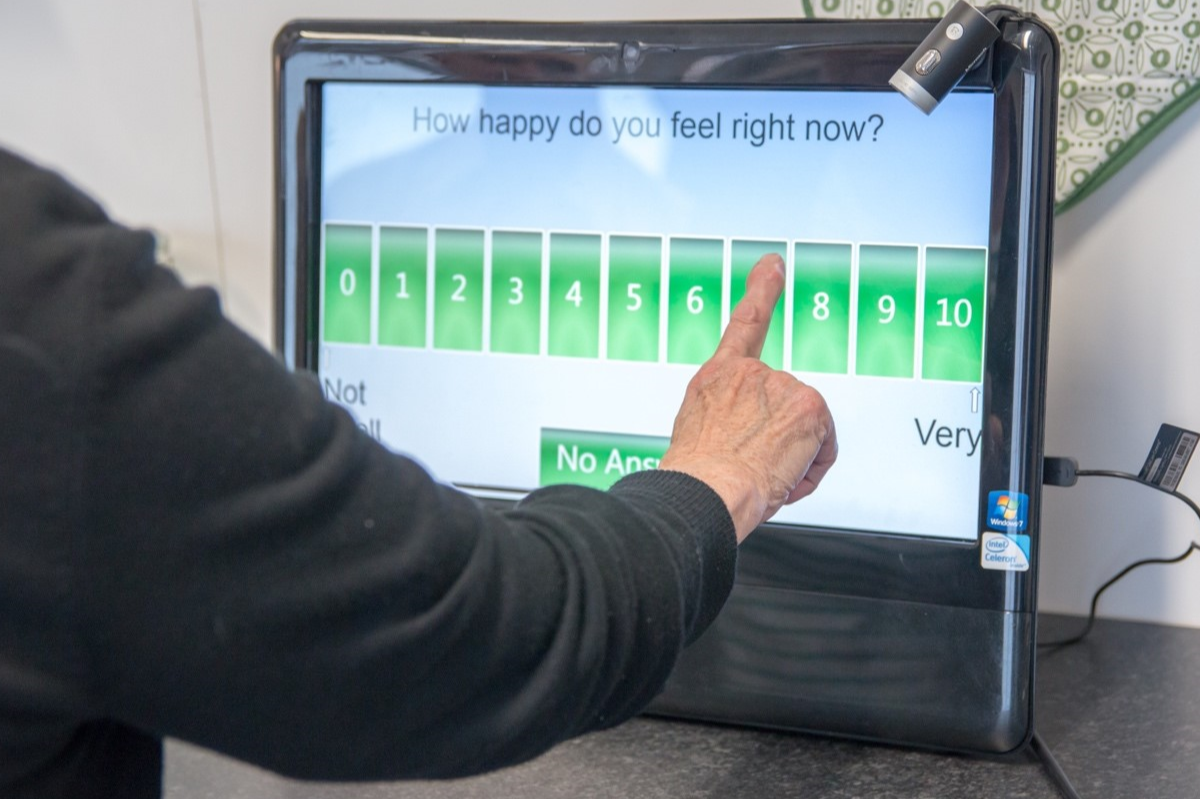
Older adult using the Novel Assessment of Nutrition and Ageing (NANA) home system on an Asus EeeTop PC.

Materials associated with each of the interactive activities are summarized in Table 1. Materials included printed and laminated cards as well as apps presented on two iPad Air devices with 9.7-inch touch screens, one Samsung Galaxy Tab 2 tablet computer with 10.1-inch touch screen, and one Asus EeeTop PC with 15-inch touch screen. The latter was used to demonstrate the Novel Assessment of Nutrition and Ageing (NANA) method, a research tool, which is pictured in Figure 1. In addition, at the beginning of their first session, participants completed a demographics questionnaire, requesting information on age, education, experience using different types of everyday technology, and self-perceived health status. Sessions were videotaped using a Sony HD Handycam on a tripod and were audiotaped using three Olympus digital audio recorders.

### Procedure

Four interactive group sessions were conducted with two groups of older adults. Each group attended two sessions of two hours in length. The sessions were conducted in the Home Laboratory in the Centre for Assistive Technology and Connected Healthcare at the University of Sheffield, United Kingdom. The Home Laboratory is a multipurpose space laid out as a furnished, one-bedroom apartment. This setting allows research participants to try out new technologies in a home-like environment. The facilitated sessions were comprised of seven activities in total—four in Session 1 and three in Session 2. In addition to activities where participants worked in pairs or small groups, whole-group discussions allowed participants to reflect on their experiences in the sessions.

### Data Analysis

All sessions were audio- and videotaped and subsequently transcribed verbatim by the first author (JAA). All personal identifiable data were removed at the stage of transcription. Transcripts were analyzed using template analysis [36]. Applying this form of analysis involved a number of steps, which led to the development of a final template of themes. First, a small number of a priori themes were developed, which formed the basis of the template. Initial themes for this study related to attitudes toward technology and mental health in general, as well as attitudes toward use of technology for the specific purpose of supporting mental health (see Textbox 1).

After familiarization, the data were coded using NVivo 11 PC software (QSR International), in line with the principles of template analysis. This was an iterative process that involved reading each transcript and identifying meaningful chunks of text that demonstrated participants’ views. Once identified, chunks were labeled in one of three ways: (1) they were assigned directly to one of the a priori themes; (2) they were assigned to new codes that were related to one of the themes; or (3) where the view represented was not related to any pre-existing theme, they were assigned a *floating* code. These were combined into new themes where appropriate. Themes were iteratively renamed, reorganized, and removed. This process was conducted by JAA. In order to improve the reliability of the analysis, LJEB and AJA also reviewed some of the data and took part in discussions throughout the process.

**Table 1:** Activities undertaken in the study.

Activity breakdown		Description	Materials	Purpose
**Session 1**				
	1. Icebreaker (10 minutes)	Participants introduced themselves and said something that cheers them up when they are feeling down.	N/A^a^	This activity aimed to make participants feel comfortable speaking in a group and to begin thinking about ideas associated with mood.
	2. Activity sorting (25 minutes)	Participants worked in pairs to decide whether or not they would use technology for a range of tasks, including some related to mental health and some not. They also considered what type of technology they might use to achieve these tasks.	Four packs of 10 laminated cards were used. Each card featured an activity that could be done with or without technology (eg, send a photo or research a health problem). These were written in size 55 text.	This activity aimed to begin to elicit attitudes toward use of technology to support mental health.
	Break (15 minutes)	N/A	N/A	N/A
	3. Vignettes (25 minutes)	Participants worked in small groups to read and discuss four vignettes describing the experience of anxious feelings and low mood.	Four packs of four vignette cards were used. These cards were written in size 14 text.	This activity aimed to engage participants in considering real-world situations in which technology might be used to support mental health.
	4. App interaction (25 minutes)	In small groups, participants tried multiple apps on a mobile phone (Samsung Galaxy S3) and on tablet computers (iPad Air and Samsung Galaxy Tab 2). Participants discussed how useful these were and decided if they (the participants) would use these in their own homes.	Apps selected were WellMind (Dudley and Walsall NHS^b^ Mental Health Partnership), Five Ways to Wellbeing (Somerset Public Health), and MindShift (AnxietyBC).	This activity aimed to explore motivators and barriers to the use of apps and websites for the purpose of supporting mental health.
**Session 2**				
	5. Show and tell (20 minutes)	In an activity inspired by the COBALT^c^ study [29], participants presented a piece of technology they loved and a piece of technology they had abandoned, along with reasons.	Participants’ own self-bought and self-chosen technologies were used.	This was a warm-up activity, aimed to give participants confidence and allow them to feel like experts on the topics at hand [29].
	6. App interface evaluation (70 minutes, including a 15-minute break)	In small groups, participants evaluated four different ways of self-reporting mood using different digital technologies in turn. Then, following a 15-minute break, the apps were discussed in the whole group.	Mr Mood and Pacifica were presented on Apple iPad Air devices; Five Ways to Wellbeing was presented on a Samsung Galaxy Tab 2; and NANA^d^ Mood was presented on a 15-inch touch screen Asus EeeTop PC.	This activity aimed to explore usability of apps for the purpose of supporting mental health.
	7. Imagining a future app (30 minutes)	Participants were asked to consider different ways an app might respond to low mood scores. After discussing different ideas in pairs or threes, ideas were discussed as a group. All discussions were recorded.	A flip chart and pens were used.	This activity aimed to allow participants to consider how data might be used and to understand how this may affect their motivation to use mood-reporting technology.

^a^N/A: not applicable.

^b^NHS: National Health Service.

^c^COBALT: Challenging Obstacles and Barriers to Assisted Living Technologies.

^d^NANA: Novel Assessment of Nutrition and Ageing.

***A priori* themes chosen before beginning the coding process.**

*A priori* themes:

- Attitudes toward technology in general
- Attitudes toward mental health conditions
- Attitudes toward technology for supporting mental health
- Motivators to use
- Barriers to use
- Usability

## Results

### Participant Characteristics

The participants’ ages ranged from 52 to 88 years and the mean age was 66 years (SD 8.6). The majority of the participants were female (12/15, 80%). Most had attended further or higher education (11/15, 73%). The majority of the participants (11/15, 73%) reported that they regularly used the Internet. Almost half of the participants reported that they used technology to manage a health condition (7/15, 47%), although no data were available on what type of technology they used or what condition they managed. All participants rated their health as fair (8/15, 53%) or good (7/15, 47%).

### Results of the Template Analysis

The final template comprised three themes, each with a number of subthemes (see Table 2). Each of these themes is described in detail in the following sections.

**Table 2:** Final template including themes and subthemes after coding.

Themes and subthemes		Quotes^a^ (n)
**1. Motivators to the use of technology to support mental health**		
	Using technology is preferential to “bothering” people when experiencing low mood	6
	Keeping a record of mood using apps and websites can facilitate self-awareness	3
	Playing music using technology can be beneficial to mood	11
	Games within apps can be used as a distraction from problems	6
**2. Barriers to the use of technology to support mental health**		
	Low mood may affect readiness to engage with digital technologies	4
	Fear of consequences may affect readiness to engage with technology	4
	Self-diagnosis using websites is problematic	9
	Technology is inferior to humans	11
	Older adults have some difficulties with usability of apps and websites irrespective of the mental health content	9
**3. Awareness of technology to support mental health**		
	Participants are aware of National Health Service websites as a source of information and resources	4
	Participants are aware of online meditation and mindfulness resources	5
	Awareness alone is not enough for successful use	6

^a^Number of quotes coded to each theme and subtheme. Coding was completed using NVivo 11 (QSR International).

### Theme 1: Motivators to the Use of Technology to Support Mental Health

Participants described several reasons they may be motivated to use technology to support their mental health. First, participants described feeling reticent to discuss if they were experiencing low mood with friends or family members for fear of bringing them down, too. Using technology in place of speaking to a person was seen as a potential way to prevent “bothering” others and thus to be self-reliant.

I’d be worried about passing misery on, you know what I mean.

Participant 11, female, age 66

If your computer was set up that way, if you spoke to it and said, “I’m in a low mood today, what should I do?” ...because the computer’s programmed to s...well like one of them Android things [sic], just to say, “well I suggest you go for a walk” or whatever it churns out, really. I think that’s good. Cos [because] you don’t always want to bother somebody, like these ladies have said, you don’t.

Participant 15, female, age 55

Loneliness was a particular source of concern for participants. Many recognized that loneliness was detrimental to health and mood. Participants discussed how they used technology to alleviate loneliness.

I will switch the radio on sometimes, but the visual contact of the TV makes me feel that I’m not on my own... It’s company, you see people.

Participant 15, female, age 55

Participants mentioned that they were motivated to use technology to play music because it helped to improve their mood. Participants used a wide variety of different technologies to source music, including YouTube [37].

If I want a lift, an uplift, I go and put rock and roll on, or summat [something] a bit lively.

Participant 14, male, age 75

I put music on. Put YouTube on and type in what I fancy listening to.

Participant 10, female, age 68

Yeah, I like the YouTube [sic] for all sorts. Er, but music is, does relax me.

Participant 9, female, age 69

Apps designed to support mental health were considered helpful in normalizing the experience of poor mental health. After testing apps that described commonly experienced symptoms of depression, anxiety, and stress, participants highlighted how reading these accounts provided them with a sense of normality.

It makes you feel normal, because you’re seeing it written down.

Participant 12, female, age 56

Video games were also seen as helpful, as a way to distance oneself from one’s problems and improve current mental state through distraction.

It’s if like, summat’s [something has] happened or you know, so it will take your mind if you’re concentrating on one thing. Just gives you that bit of respite.

Participant 14, male, age 65

Participants recognized these benefits in a game within an app entitled WellMind that was explored during the study session.

Oh there’s playing a game with a kind of snake and an apple, you have to move a bar and hit the apple and things...I suppose it’s a kind of concentration of the mind.

Participant 12, female, age 56

### Theme 2: Barriers to the Use of Technology to Support Mental Health

Participants also recognized a number of barriers to the use of digital technology to support mental health. First, participants suggested that low mood was likely to affect readiness to engage with digital technologies.

I do wonder if you could be so low, that you couldn’t be bothered to go on a computer.

Participant 11, female, age 66

Participants also commented that the fear of the consequences of using technology to support mental health may also prevent them from engaging with mood-reporting tools, such as apps, to support mental health.

Where the fear is of something unpleasant happening to you, like you’re being taken, taken into an institution, then you’re not going to admit any symptoms that you might think could be interpreted that way.

Participant 8, female, age 68

Some participants mentioned that using technology to self-diagnose could be problematic, since it could result in patients mistakenly believing they had a rare or extreme medical condition.

To research online a specific problem, you know, say diabetes, is ok, but to research and put in what problems you’ve got that come up with some outlandish thing is wrong. I don’t agree with that. The doctor tells you that.

Participant 6, male, age 69

As well as these concerns, there was a sense that human contact was superior to technology when it comes to mental health and that dealing with people was preferable to using technology.

If you really feel down and you really need some help, can a computer...don’t you think physical contact or speaking to someone is far more important?

Participant 13, female, age 60

Some technology usability issues were discussed, which, while not specific to apps to support good mental health, nonetheless created barriers to successful engagement. Many of the apps evaluated by participants assumed a certain level of prior knowledge on the part of their users. For example, some apps use a cross (X) symbol on buttons to close a text-entry box, while others used a swipe action to move between options. For some participants, these assumptions caused difficulties, as they did not always have the required prior knowledge, skills, or experience.

If you push that cross does that mean it cancels it out? Yes. Right. You do it.

Participant 12, female, age 56

No I think it, does it, oh.

Participant 13, female, age 60

Dexterity was also an issue raised by several participants. One of the apps tested by participants was Pacifica. This app featured a circular slider for users to report on how they were feeling. Participants suggested this action would be particularly difficult for someone who had had a stroke.

I think, if they’ve come out of hospital and had a stroke, that turning one [ie, Pacifica] might be quite difficult for them.

Participant 15, female, age 55

They also suggested that poor dexterity may be a barrier to the use of games within apps to support mental health.

I think that you’ve...that snake game, your hands have to be quite good.

Participant 12, female, age 56

Yeah, dexterous.

Participant 11, female, age 66

Yeah, your dexterity has to be pretty good for that one.

Participant 12, female, age 56

Further, usability-related barriers found in this study included difficulties reading small fonts; for example, Participant 15 stated, “The print’s too small, can you make it bigger?” There was also a sense that using technology is effortful and time-consuming; for example, Participant 2 stated, “When you go on a computer, you’ve got to log on and all that blah, blah, blah.” Some participants also mentioned that they experienced fear when completing certain activities on digital platforms; for example, Participant 13 stated, “I don’t know why I’m afraid of downloading apps.”

### Theme 3: Awareness of Technology to Support Mental Health

Participants demonstrated an awareness of multiple uses of digital technologies to support mental health. These included online doctor’s appointment booking systems, National Health Service (NHS) and other health websites for researching health conditions, meditation apps, and YouTube videos for relaxation. One participant expressed confidence using the NHS website and saw the potential benefits of using it. Here, she reacts to a vignette in which the person described is imagined to be experiencing symptoms of depression.

I suppose for this one, this red one, you could like, look up a...NHS site, Moodzone, they do audio tapes that, they do tapes that actually give you some inspiration towards feeling better.

Participant 13, female, age 60

Many participants referred to CDs, tapes, and online resources for mindfulness, though one participant was unsure about digital delivery.

How does mindfulness work online?

Participant 1, female, age 66

Another participant found it difficult to use such resources alone.

I’ve got those tapes that they’ve given me to teach me how to relax. I find that, I can’t do it on me [sic] own. But I can do it say if you was [sic] with me, and we just, meditated together, I could do that. But on my own, I’m finding, “oh I haven’t washed the pots, I haven’t done this, I haven’t done that,” you know.

Participant 15, female, age 55

Thus, although participants showed an awareness of various ways in which digital technologies can be used to support mental health, awareness alone may not be enough for participants to start using such tools successfully. Indeed, not everyone was convinced there is a role for technology in certain situations.

I don’t know how technology would help.

Participant 9, female, age 69

## Discussion

### Principal Findings

This study used the COBALT principle of user as expert to understand the motivators and barriers to older adults’ use of technology to support mental health. New findings presented in this work include that self-reliance, averting loneliness, normalization, facilitated expression, and improving mood can be motivators for older adults to use technology to support their mental health. Some apps and websites included in the study were found to offer functions that assisted users in understanding their mental states and in facilitating behaviors that would promote well-being, flourishing, and mental health. The study has also illuminated possible reasons older adults might avoid using technology to support mental health, including low mood itself, fear of consequences, and a preference for human contact.

The participants expressed an interest in using technology to alleviate low mood, for example, by listening to music on YouTube or playing games within apps. They expressed how playing games, such as that within the app WellMind, could be a helpful distraction from unwanted thoughts and may help to concentrate the mind. Prior research has highlighted that technology can provide older adults with opportunities for enjoyment and fun [22]. This study extends this to the added benefit of alleviating low mood and providing distraction. Older adult participants also highlighted how the framework provided by mood-reporting tools within apps could be helpful for exploring their own feelings and being able to express these. Furthermore, reading descriptions of the experience of symptoms of poor mental health was felt to be normalizing. Our findings suggest the inclusion of elements such as the ability to play music, play games, read about symptoms, and report on mood within an app to support mental health is likely to be motivating and beneficial for older adult users and could promote flourishing, as described by Keyes [10]. However, further research would be required to ascertain the true level of benefit each of these elements could provide.

Findings here reflect how older adults may turn to technology to manage symptoms of low mood and alleviate feelings of loneliness by themselves, without reaching out to friends or family. Participants described how using apps to record their mood and receive activity suggestions may be preferable to speaking with others when experiencing low mood, to avoid being seen as a burden. Peek et al [28] also found that older adults were keen to avoid being a burden and used technology to this end. The determination of older adults to be self-reliant can thus be seen as a motivator for them to engage with technology to support their mental health. However, some participants felt that, if affected by a mental health condition, they would prefer to speak with a human about their experience rather than use technology. Views on the superiority of humans over technology thus contrasted with views around the benefits of technology when an individual did not feel confident speaking with a person. These differing views on the best way to support mental health demonstrate there is no “one-size-fits-all” in the use of digital solutions to support mental health and that there are situations in which technology can be seen as having advantages over human contact. Prior work has also highlighted both the preference of older adults for human management of health care [38] and, contrastingly, the benefits of using technology to support health care in order to avoid being a burden on others [28]. This tension could be resolved in future applications of digital technology for the management of older adults’ health and well-being through a decision aid to help decide whether contact should be made with a health professional and/or a formal or informal carer.

The participants identified a number of barriers to using technology to support mental health. Discussions revealed how symptoms of poor mental health may affect readiness to engage with technology. Comments included the fact that feeling depressed was likely to magnify difficulties with usability of digital technologies. The Diagnostic and Statistical Manual of Mental Disorders lists lack of motivation and reduced pleasure in daily activities as symptoms of major depression [3] and participants’ comments in this regard are thus in line with these symptoms of depression. Further research would be required to understand this phenomenon more fully, for example, exploring what severity of depression or anxiety causes disengagement with technology and in what populations this is most common. The use of prompts, either originating from the devices themselves or from family members or health care practitioners, could also be explored as a potential method to mitigate this disengagement.

Some usability issues with the apps and websites were identified. For example, the use of interactive COBALT activities revealed that some participants struggled to understand the meaning of relatively standard symbols and dialogues within apps and websites. This suggests that apps and websites must address accessibility and universal design features to maximize their usability and reach to all potential beneficiaries, rather than assuming prior knowledge. Similar findings were reported by Eisma et al [39], who demonstrated that scrollbars were not familiar to older adults, and Grindrod et al [40] who discussed how “Cancel” buttons, scrolling functionality, autocorrect, sample text in grey fonts, and peripheral buttons can confuse older adult users with less experience using technology. Vaportzis et al [41] reported on older adults’ struggle to use digital technologies where physical health complaints, such as poor eyesight or limited dexterity, affected interaction with devices. Our findings support these points and further demonstrate the need for lower dexterity to be kept in mind when developers are designing apps for older adults. Thus, to ensure older adults feel comfortable and confident using digital technologies to support their mental health, apps and websites should use intuitive layouts with easy-to-use function buttons and clear explanations, while avoiding the use of technical jargon.

Another perceived barrier to using technology to support mental health was the perception that using digital technology might result in automated decisions about treatment being taken. When discussing the use of digital technology to report on mood, participants suggested that fear of being institutionalized could affect honesty in self-reports. Prior work has indicated the benefits and psychological attachments older adults have to ageing in their own homes [42,43]; the findings in our study indicate that the possibility of losing this independence could provoke anxiety for older adults using digital technology to monitor mental health. This finding could inform the development of documents and guidance provided alongside apps and websites to address users’ fears about the implications of using the technology. For example, these materials might explain that these technologies are designed to be informative rather than diagnostic and that their use would not lead to any automatic decisions about their future care or treatment being made.

### Limitations

Although this study has produced novel insights into older adults’ attitudes toward the use of technology to support mental health, it does have some limitations. First, there were few participants with very low levels of education or very limited experience of technology, meaning results may reflect a greater awareness of, or ability with, technology among participants than is representative of the population at large. Second, the majority of participants in this study were female, meaning views of older males may be underrepresented. Third, while the inclusion of participants aged 50 and over enabled us to gain a more diverse range of perspectives, it also means that not all points may be relevant to all age groups. Future studies that examine the relevance of each theme to more homogenous subgroups of older people would therefore be useful for learning more about the different types of potential users of mental health technologies.

### Conclusions

This study has revealed that older adults are motivated to use digital technology to support their mental health, in particular to promote self-reliance, avert loneliness, and improve mood. However, low mood, fear of consequences, and a preference for human contact may prevent successful engagement with these technologies. Further work is needed to understand how the use of music and games within apps and websites might help to address symptoms of mental illness in older adults and to understand how different states of mind, such as low mood, might affect older adults’ readiness to engage with technology. Developers of apps and websites designed to support mental health that could be used by older adults should be mindful that technical jargon and commonly used symbols may not be understandable to all older adults. They should also be aware that guidance provided with such tools could be helpful in reassuring users that using these tools will not reduce human contact with health professionals or result in adverse consequences.

## Abbreviations

COBALT: Challenging Obstacles and Barriers to Assisted Living Technologies
NANA: Novel Assessment of Nutrition and Ageing
NHS: National Health Service
